# Sacral Neuromodulation in Pregnant Women—A Case Report and Literature Review

**DOI:** 10.3390/ijerph19148340

**Published:** 2022-07-08

**Authors:** Jacek K. Szymański, Aneta Słabuszewska-Jóźwiak, Grzegorz Jakiel

**Affiliations:** First Department of Obstetrics and Gynecology, Centre of Postgraduate Medical Education, ul. Żelazna 90, 01-004 Warsaw, Poland; anetaslabuszewska@gmail.com (A.S.-J.); grzegorz.jakiel1@o2.pl (G.J.)

**Keywords:** pregnancy, overactive bladder, urinary retention, sacral neuromodulation

## Abstract

Millions of women around the world suffer from an overactive bladder and urinary retention. A significant number of them are of reproductive age. For 25 years, SNM has been an effective therapy for treatment-resistant hyperactive bladder and idiopathic urinary retention. The paper presents a case of a 35-year-old pregnant woman with an overactive bladder resistant to pharmacological treatment, who responded positively to sacral neuromodulation. The patient decided against deactivating the neuromodulator and, after an uneventful course of pregnancy, she gave birth by a caesarean section to a healthy female infant. The use of SNM in pregnant patients remains a constant clinical challenge. The current literature was reviewed, but published studies do not provide a clear answer. Further studies with a long follow-up period are necessary to determine more accurately the effects of SNM therapy on the fetus and the course of pregnancy. Currently, it is recommended to deactivate SNM during pregnancy. However, it seems that an individual approach to the patient with information on the risks and benefits of continuing or discontinuing therapy should be the current procedure.

## 1. Introduction

Urinary tract dysfunctions affect millions of women around the world. Epidemiological studies reveal the presence of an overactive bladder in 16% of women in the US and 17% in Europe [1,2]. The overactive bladder (OAB)-POLL survey and the Epidemiology of Lower Urinary Tract Symptoms (EpiLUTS) studies revealed an even higher OAB prevalence of 29.4 and 36.2%, respectively. The studies showed an increasing trend in OAB symptoms with age; however, it should be considered that a significant number of the women suffering from this pathology were in the reproductive time of their life [3,4]. Contrary to this, urinary retention that could be related to detrusor underactivity mostly affects post-menarche young women in the second and third decades of life [5,6]. Introduced in the eighties, sacral neuromodulation (SNM) has been an effective therapy for the treatment of a refractory hyperactive bladder and idiopathic urinary retention in selected patients [7,8]. Therefore, the possibility of pregnancy in patients treated with this method should be considered. If this happens, the patient and the obstetrician are faced with a difficult decision of whether to turn off the device or continue operation. The questions arise of what is the potential effect of sacral neuromodulation on the course of pregnancy: does it affect the development of the fetus, and what effect will turning off the device and the potential recurrence of lower urinary tract symptoms (LUTS) have on the course of pregnancy?

## 2. Aim

This study aims to present a case of a 35-year-old woman who decided to keep the SNM active throughout the whole pregnancy and to review the literature on the use of sacral neuromodulation in pregnancy.

## 3. Case Report

We present the case of a 35-year-old woman with a refractory overactive bladder. The patient was unsuccessfully treated with anticholinergic drugs, mirabegron, and three injections of botulinum toxin in the bladder. The micturition diary showed 13 micturitions per day with both urgency and involuntary leakage of urine, and 4 episodes of nocturia. The patient also presented 14 points according to the Overactive Bladder Symptom Score (OABSS) [9]. Several days after implantation, the patient reported a reduction in daily voids to 7 in the absence of urgency and a reduction in the number of nocturia to 0–1. In addition, there was also an improvement expressed in the reduction in all OABSS domains with a final score of 2 points.

After such a positive response to the first stage of sacral neuromodulation, the patient was implanted with a permanent implantable pulse generator (IPG) in the buttock. One month after implantation, the patient became pregnant. Immediately after the diagnosis of pregnancy, an interview was carried out with the patient regarding the decision to keep the device working or to deactivate it. Based on the sparse literature, the patient was presented with the current state of knowledge so that she could make an informed decision. The patient refused to turn off the device even for the first trimester of pregnancy, justifying her decision with the fear of the recurrence of OAB symptoms. This decision could also have been influenced by the short interval between the implantation of the neuromodulator and achieving pregnancy, since the patient remembered the spectrum of symptoms that made her life difficult.

The course of the pregnancy was uneventful. The first trimester ultrasound did not show any increased risk of genetic defects in the fetus. Similarly, the ultrasound examinations in the second and third trimesters of pregnancy did not reveal any congenital abnormalities of the baby. Active neuromodulation allowed the patient to keep control of voiding. She did not report any adverse effects of electrical stimulation. At full term, she gave birth by caesarean section to a 3.320 g healthy female with an Apgar score of 10. The caesarean section was performed for psychological reasons. The patient was very concerned about the potential risk of lead displacement and damage to the IPG during a vaginal delivery. The SNM was deactivated during the caesarean section and reactivated 4 h after surgery. The course of the caesarean section and spinal anaesthesia was uneventful. There was no disturbance in the work of the neuromodulator in the postpartum period. The patient persists with the outpatient follow-up schedule consistently, with a persistently good response to sacral neuromodulation (OABSS remains at 2–3 points).

## 4. Materials and Methods

A literature search was performed in December 2021 using the Medline and Embase databases with search results from 2005 to 2021. The adopted criteria allowed for the inclusion of studies on humans and animals, original papers, case reports, and literature reviews limited to those published in English. Studies on the safety and effectiveness of neuromodulation in pregnancy were identified using various MeSH headings. The following phrases were used: “pregnancy and urinary retention”, “pregnancy and overactive bladder”, and “pregnancy and sacral neuromodulation”.

## 5. Results

A total of 347 articles were identified. After excluding duplicates, articles only available as abstracts, articles in languages other than English, and articles not related to sacral neuromodulation therapy, only 11 papers remained. Three original animal studies [10,11,12], six case reports [13,14,15,16,17,18], one systematic review, and one literature review [19,20] were analysed. The case reports included 1 to 14 pregnancies in women treated with SNM. The relevant findings are summarised in Table 1.

## 6. Discussion

An overactive bladder and nonobstructive urinary retention affect a fair number of women of childbearing age. Many of them were put on sacral neuromodulation; however, a possible pregnancy could limit this kind of therapy. Due to insufficient data and the lack of consensus among experts, it is currently recommended to deactivate the device during pregnancy [21].

### 6.1. Animal Studies

The concern was raised about the hypothesised effects of SNM treatment during pregnancy including teratogenicity, fetal malformations, abortion, premature labour, irritation, ulceration of the stretched skin over the battery, anaesthetic care difficulties, pain at the lead site, lead migration, battery failure, and stretching the lead extender by the expanding abdomen. None of these adverse effects were seen in animal studies. Contrarily, the electrical stimulation of pregnant rats and rabbits revealed a long-lasting inhibitory effect on uterine contractility during pregnancy and labour. Moreover, one of the continuous modes of electrical stimulation in pregnant rats that were induced to deliver preterm resulted in lower intrauterine pressure, increased delivery time at term, and delayed delivery in preterm birth. Additionally, such inhibitory actions did not correlate with systematic changes in the pregnancy hormonal state [10,11]. The courses of pregnancies of the stimulated rats were uneventful, with no fetal anomalies or abortions [12].

### 6.2. Safety of Conception

The lack of well-designed prospective studies, the relatively small numbers of patients in case series, and the heterogeneity of case reports results in many unanswered questions concerning the safety of SNM in pregnancy. Nevertheless, several conclusions can be drawn from the published articles. The safety of conception was investigated by Yaiesh and colleagues in a literature review [20]. The authors did not find any adverse effects of low-frequency electromotive forces on fertility. Moreover, they concluded that the reduction of urological symptoms followed by an improvement in the quality of life could result in better sexual health, increasing the chances of conceiving. Theoretically, SNM activity, due to the presence of a pelvic electromagnetic field, may be teratogenic, especially in the first trimester of gestation, and responsible for early or late miscarriage [22]. The reviewed cases did not reveal any fetal abnormalities or abortions that could be solely attributed to pregestational neuromodulation or the presence of a device implanted during pregnancy, even in the cases that continued with neuromodulation throughout gestation. Of the two newborns in the cases mentioned above, one developed a motor tic and the other was born with a pilonidal sinus, but these probably cannot be directly linked to neuromodulation. Motor tics are possibly genetically determined; however, epigenetic, autoimmune, and environmental factors are also involved in their aetiology [23]. Therefore, SNM as a possible cause, although unlikely, cannot be definitely excluded. Pilonidal sinus is most often congenital; however, the aetiology of this disease is not clear [24]. In addition, the patient developed gestational diabetes and kept the IPG inactive during pregnancies. Similarly, the two cases of miscarriage were probably related to an increased risk of abortion in an IVF pregnancy and a general risk of abortion in early pregnancy, respectively. Moreover, the second patient later went on to have an uncomplicated pregnancy and delivered a healthy child despite keeping the SNM device on. Of note, she switched the device off in her first pregnancy, thus reducing the direct impact of SNM on a miscarriage [20]. It is plausible that the electromagnetic field generated by the implanted IPG could penetrate soft tissue, muscle, and the iliac crest to affect the fetus. However, the animal studies that were carried out concluded no adverse effects of low-frequency on teratogenicity and fertility [17].

### 6.3. Impact on Pregnancy Duration

The possible impact of SNM on preterm labour should also be taken into consideration. The contractile activity of the pregnant uterus is dependent on a complex system of hormonal and non-hormonal inhibitors. According to Bernardini and Karsdon [10,17], there is no effect of SNM on the systemic levels of reproductive hormones, and sacral neuromodulation does not affect the maintenance of the gravid uterus, nor does it play a role in the initiation of labour. Furthermore, SNM could cause vasodilatation; therefore, it is unlikely to have any adverse effects on uteroplacental blood flow. On the contrary, as it was mentioned above, the deactivation of the device resulted in a recurrence of urinary symptoms, such as urgency and urinary retention, complicated by UTIs. In turn, UTIs put pregnancy at risk for preterm labour [25,26]. The correlation between neuromodulation and uterine contractile activity seems to be questionable. Although animal studies have shown an inhibitory effect of current stimulation on uterine contractions, it seems that too few human observations do not allow for a definitive explanation of this relationship.

Of the 34 analysed pregnancies, 13 that kept the neuromodulator active and 12 that deactivated the device delivered on term. Of patients who gave birth prematurely, 1 maintained neuromodulation and 8 turned off the IPG. The causes of preterm labour were various: premature uterine contraction, premature detachment of the placenta, recurrent UTIs, chronic symphysis pubis dysfunction, and the need for chemotherapy for breast cancer [13,16].

### 6.4. Complications with Keeping the Device On/Off

Several human case reports and retrospective case series were published; however, the number of reported cases is not large. Although the patients were advised to stop neuromodulation during pregnancy, some of them decided to continue the treatment. They were faced with putting the development of the fetus and the course of pregnancy at risk to prevent the signs and symptoms from before the treatment returning. Switching the neuromodulator off could potentially result in urinary frequency, urge urinary incontinence, or urinary retention with self-catheterisation and a risk of urinary tract infection. Khunda and colleagues reported a series of 13 pregnancies in 10 patients with Fowler’s syndrome [13]. The pregnancies were diagnosed between the fourth and sixth week of gestation in all cases. The implantable pulse generator (IPG) was turned off at 4–12 weeks of gestation in seven pregnancies, at the beginning of the second trimester in two, and kept on against medical advice in three. After turning off the SNM, voiding dysfunctions and chronic urinary retention recurred in nine out of ten pregnancies, complicated by urinary tract infections in four cases. Severe pyelonephritis occurred in one woman. Seven women reached full-term pregnancy, four of whom delivered by spontaneous vaginal route, one with a forceps delivery, and the remaining three had a caesarean section performed for obstetric indications. Five patients whose pregnancies ended preterm underwent a caesarean section. The average birth weight of the infants was 3.067 g, and no congenital abnormalities were reported. The three women who kept the SNM on had an uneventful pregnancy and continued to void normally after delivery. Switching the SNM back on resulted in the same effectiveness as before the pregnancy in five cases, but failed in four. Ansó and colleagues reported a case of a 16-year-old woman with Fowler’s syndrome who was successfully treated with an SNM before pregnancy [14]. The pregnancy was diagnosed at 10 weeks and the neuromodulator was switched off according to the FDA and the manufacturer’s advice. The patient developed urinary retention complicated by a urinary tract infection. She delivered a healthy baby at term with successful neuraxial analgesia. The other case of pregnancy in a woman treated with neuromodulation due to an electrical spinal cord injury was reported by Mamopoulos and colleagues [15]. Here, the patient decided against deactivating the device, fearing recurrent urinary infections caused by having to self-catheterise. The course of pregnancy was uneventful with no signs of a threatened preterm labour. She voided normally and urinary infections were not detected. At 39 weeks of gestation, she delivered a healthy male infant by caesarean section due to a breech presentation. Sacral neuromodulation was active throughout the pregnancy and postpartum, and no issues were raised regarding the malfunction or displacement of the device. Subsequently, Agnello and colleagues [16] reported a case series of 14 pregnancies among 11 women with urinary retention and dysfunctional voiding treated with SNM. Two patients deactivated the devices from the first trimester and ended up having to return to self-catheterisation and experiencing recurrent UTIs. The remaining nine patients kept the SNM on during pregnancy; however, two of them tried to temporarily switch the device off in the second and third trimester. Due to the recurrence of urinary retention, they turned the neuromodulator on and once again achieved spontaneous micturition. One of the nine patients experienced perineal paraesthesia with no need to decrease the amplitude of stimulation. Eleven pregnancies were resolved with a caesarean section (four due to obstetric indications, and seven to avoid lead displacement during spontaneous delivery) performed under regional anaesthesia, except for one general anaesthesia. Three pregnancies ended in a vaginal delivery. In one case, after childbirth, the device had to be removed due to loss of efficiency. No congenital abnormalities were observed in newborns and no lead displacements were reported.

The effect of SNM on pregnancy was analysed in a systematic review including 26 pregnancies in 22 women. A neuromodulator was implanted for Fowler’s syndrome in 14 pregnancies, and for urine retention in 6 women. The other reasons included faecal incontinence, urinary urgency and frequency, faecal and urinary urgency, intractable interstitial cystitis, and myelodysplasia. The device was deactivated in 18 pregnancies, resulting in recurrent urinary tract infections. Two patients requested SNM reactivation at 19 and 20 weeks of gestation for reasons of urinary retention and faecal and urinary urgency symptoms. The only pregnancy that resulted in a miscarriage was an IVF pregnancy. There were 18 full-term deliveries versus 7 preterm. In total, 16 pregnancies ended by caesarean section (mostly due to obstetric reasons), whereas 9 had vaginal deliveries, of which 2 were assisted with the use of forceps or a vacuum. Out of 25 newborns, 2 (delivered by the same mother) had pilonidal sinus and motor tic disorders. Postpartum, the SNM was effective in 14 patients, whereas 3 required reprogramming, 1 required revision, 3 required replacement, and 4 decided on the removal of the device [19]. Yaiesh and colleagues profoundly reviewed several case reports and series describing continued SNM, as well as other related modes of neuromodulation during gestation [20]. They found no negative effects of SNM on the fetus and the mother. These observations were confirmed by a national survey carried out for the neurology committee of the French Association of Urology [27]. In total, 27 pregnancies among 21 women were recorded. The median time between implantation and the first pregnancy was 2.6 years. In five pregnancies, the device had been turned off whilst trying to conceive and it had been disabled during the first trimester in all remaining pregnancies (with a median switch-off time of 5.5 weeks of gestation). Three of the four women with chronic urinary retention resumed self-catheterisation, and six pregnancies developed symptomatic tract infections. The 27 pregnancies resulted in 24 live births, 1 spontaneous miscarriage, and 3 voluntary interruptions of pregnancy. Twenty-six pregnancies ended at term and a caesarean section was performed for obstetric reasons in four cases. One case of premature birth was noted in women treated for an idiopathic disorder of the filling phase of the bladder. No neonatal problems were reported. The women reactivated their devices at a median time of four weeks postpartum in 20 cases and a reduction in the efficacy of the SNM was observed in 4 cases. The loss of efficacy was linked to a displacement of the electrode in two women (one of whom had had a caesarean section and one a vaginal delivery); in the remaining two patients, no specific cause was identified. The effect of SNM on fetal heart monitoring is not well documented and the only information that has been brought forth is a case of a patient with sacral cord stimulation (SCS) that remained active throughout the whole pregnancy and labour, which was reported by Bernardini [17]. No unusual activity of the fetal heart was observed. The low electromotor force generated by the neurostimulator probably has no possibility of interfering with the cardiotocography (CTG) electrodes.

### 6.5. Mode of Delivery

Other concerns have been raised concerning the mode of delivery and potential anaesthetic care during labour. Bernardini [17] reported no conflict between SCS placed in a rostral fashion at the thoracolumbar junction of the spine and spinal or epidural analgesia. It seems reasonable that the electrode of the SNM that stimulates the sacral roots should have no impact on anaesthesia during labour and, conversely, anaesthetic procedures are unlikely to cause lead migration. Nevertheless, the anaesthetic technique should include the entry point and path of the electrodes to the epidural space, and the location of the impulse generator and its intermediate connections to avoid displacement or damage with an epidural needle or catheter [14]. The mode of delivery should be the subject of discussion between the urogynaecologist in charge of device maintenance, the obstetrician, the anaesthetist, and the patient to prepare a suitable plan of care. It should also factor in the possible damage of the electrode during delivery and labour. The pregnancy itself puts the proper functioning of SNM at risk due to the changes in the lumbar spinal curvature and the pressure exerted by the gravid uterus. There are no clear recommendations for the mode of delivery. During a vaginal delivery, the maternal position, pressure from the fetal head, and the effort of pushing can theoretically directly displace the SNM electrode [13,18]. Interestingly, Khunda and colleagues observed higher rates of device dysfunction among women who delivered by caesarean section (38%) compared to those who delivered naturally (25%) and were advised a C-section for an obstetric reason only [13]. Additionally, an abdominal placement of an IPG can increase the risk of neuromodulator damage during a caesarean section. The stimulator should be switched off for a caesarean delivery due to the risk of surgical diathermy or electrocautery and the risk of damaging the stimulator by temporary suppression or reprogramming, as well as for reasons of possible electric shock in the patient [14]. As there is no convincing evidence that a caesarean section protects against lead displacement and IPG damage, it seems reasonable to perform a caesarean section solely for obstetric indications. In the presented case, the caesarean section was carried out because the patient’s fear of a potential disturbance of the neuromodulator’s function was so great that she did not consent to a vaginal delivery. After delivery, SNM efficacy must be checked to exclude displacement or electrode damage. Roulette and colleagues reported that SNM efficacy deteriorated in 20% of women postpartum, irrespective of the mode of delivery [27]. In most of these cases, the reprogramming of the device was sufficient to restore its proper functioning, with explantation hardly ever being required. The women who kept the SNM on throughout their pregnancy continued to void normally [13]. There are also reports of patients who no longer required SNM treatment after delivery, regardless of whether or not their symptoms were controlled during pregnancy without the device. This phenomenon could result from a combination of hormonal effects, pelvic accommodation during pregnancy, and neural circuit re-education [20].

### 6.6. Efficacy of SNM after Delivery

The potential for damage or displacement of the electrode during pregnancy and childbirth, and the long period of inactivity of the device before restarting it, raises concerns about the loss of therapeutic efficacy of SNM after delivery. Among the analysed cases, 27 correctly maintained their operation. Although no lead displacement or device damage was observed in any of the cases, in 6 patients, restarting the neuromodulator did not reduce symptoms. In four patients, reprogramming of the device was sufficient to restore proper operation, whereas in two cases the device had to be explanted due to its ineffectiveness. In one woman, the device was not restarted on purpose because she had properly controlled voiding during pregnancy despite the SNM being turned off [13,16]. Considering such sparse data, it seems that pregnancy and delivery do not pose a high risk of loss of SNM efficacy.

## 7. Conclusions

The decision-making process as to whether the device should be switched off or kept on requires an individual approach for the patient. The patient must be informed about the potential theoretical teratogenic and abortifacient effects of a low-frequency electromagnetic field, although—to date—no SNM-attributable fetal complications have been reported. She must also be made aware that the deactivation of the device increases the risk of the recurrence of symptoms, such as chronic urinary retention requiring self-catheterisation, with its associated higher frequencies of urinary tract infections, as well as urinary frequency, urgency, and pelvic pain. The potential advantage of the inhibitory effect on uterine contractions in the prevention of preterm labour may turn out to be disadvantageous by extending the time of delivery at term. More studies with long-term follow-up are warranted to evaluate better the risk of this therapy on the fetus and the course of pregnancy. For now, in all cases, a profound risk–benefit analysis should be performed, and the possibility of keeping the neuromodulation active should be considered with caution. Each pregnant woman treated with SNM, regardless of whether the device is active or deactivated, should be included in the group of high-risk pregnancies and be under specialist care.

## Figures and Tables

**Table 1:** Summary of the included studies. SNM—sacral neuromodulation, SCS—spinal cord stimulation, CPRS—complex regional pain syndrome, OAB—overactive bladder, CUR—chronic urinary retention, rUTIs—recurrent urinary tract infections, IVF—in vitro fertilization, CS—cesarean section, SVD—spontaneous vaginal delivery.

Study	Number of Pregnancies	Type of Neuromodulation	Indication for Treatment	Course of SNM in Pregnancy	Complications	Delivery Mode	Fetal Abnormalities	Device Sustainability
Khunda et al., 2013 [13]	13	SNM	Fowler’s syndrome	10—off (1 before IVF procedure, 7 in 1st trimester, 2 in 2nd trimester) 3—on	1—miscarriage (IVF) 9—CUR 4—rUTIs Not reported	Preterm: 5—CS At term: 1—CS, 2—SVD, 1—forceps At term: 2—CS, 1—SVD	Not observed Not observed	5—yes 4—no 1—device not restarted (normal voiding) yes
Anso et al., 2017 [14]	1	SNM	Fowler’s syndrome	Off in 1st trimester	CUR, rUTIs	SVD	Not observed	yes
Mamopoulos et al., 2014 [15]	1	SNM	Chronic urinary retention	On throughout	Not reported	CS	Not observed	yes
Agnello et al., 2021 [16]	14	SNM	7—urinary retention 4—dysfunctional voiding	5—off in 1st trimester 2—off temporarily in 2nd and 3rd trimester 7—on throughout	CUR rUTIs CUR 2—UTIs (correct bladder emptying) 5—not reported	Preterm: 1—SVD At term: 1—SVD 3—CS Preterm: 2—CS Preterm 1—CS At term: 1—SVD 6—CS	4—Not observed 1—Psychomotor developmental delay Not observed Not observed	2—no (1 removal) 3—yes yes yes
Bernardini et al., 2010 [17]	3	SCS	3—CPRS	2—off in 1st trimester 1—off in 1st trimester/on in 3rd	3—Pain	1—SVD 2—CS	Not observed	yes
El-Khawand et al., 2012 [18]	2	SNM	Bladder Pain Syndrome	2—on	Not reported	2—CS	1—motor tic (at 2 years of age) 1—pilonidal sinus	yes
Mahran et al., 2017 [19]	26	SNM	14—Fowler’s syndrome 1—fecal incontinence 1—OAB 1—fecal and urinary urgency 6—urinary retention 2—interstitial cystitis 1—myelodysplasia	15—off in 1st trimester 4—off before conception 5—on	1—miscarriage (IVF) 17—CUR, rUTIs 2—not reported Not reported	Preterm: 6—CS At term: 5—CS 7—SVD 1—forceps 1—vacuum At term: 5—CS	Not observed 1—motor tic 1—pilonidal sinus 3—not observed	14—yes 3—reprograming 1—revision 3—replacement 4—removal (2-asymptomatic; 2-dyssatisfied)

## Data Availability

Not applicable.
